# Talking Ethics Early in Health Data Public Private Partnerships

**DOI:** 10.1007/s10551-023-05425-w

**Published:** 2023-05-18

**Authors:** Constantin Landers, Kelly E. Ormond, Alessandro Blasimme, Caroline Brall, Effy Vayena

**Affiliations:** 1 Health Ethics and Policy Lab, ETH Zurich, Hottingerstrasse 10, 8032 Zurich, Switzerland; 2 Ethics and Policy Lab, Multidisciplinary Center for Infectious Diseases, University of Bern, Länggassstrasse 49a, 3012 Bern, Switzerland; 3 ELSI Advisory Group, Swiss Personalized Health Network, Laupenstrasse 7, 3001 Bern, Switzerland; 4 Institute of Philosophy, University of Bern, Länggassstrasse 49a, 3012 Bern, Switzerland

**Keywords:** Data sharing, Stakeholders, Ethical issues, Public private partnerships

## Abstract

Data access and data sharing are vital to advance medicine. A growing number of public private partnerships are set up to facilitate data access and sharing, as private and public actors possess highly complementary health data sets and treatment development resources. However, the priorities and incentives of public and private organizations are frequently in conflict. This has complicated partnerships and sparked public concerns around ethical issues such as trust, justice or privacy—in turn raising an important problem in business and data ethics: how can ethical theory inform the practice of public and private partners to mitigate misaligned incentives, and ensure that they can deliver societally beneficial innovation? In this paper, we report on the development of the Swiss Personalized Health Network’s ethical guidelines for health data sharing in public private partnerships. We describe the process of identifying ethical issues and engaging core stakeholders to incorporate their practical reality on these issues. Our report highlights core ethical issues in health data public private partnerships and provides strategies for how to overcome these in the Swiss health data context. By agreeing on and formalizing ethical principles and practices at the beginning of a partnership, partners and society can benefit from a relationship built around a mutual commitment to ethical principles. We present this summary in the hope that it will contribute to the global data sharing dialogue.

## Introduction

The sharing of health data among stakeholders in a trustworthy manner is a key challenge of innovation in health, particularly in innovative fields such as digital health or personalized health (Krumholz, 2015; Milham, 2018; Vayena et al., 2018). As personalized health relies on the aggregation, analysis, and interpretation of diverse health and other datasets, data sharing is vital to realizing its promise (Hulsen et al., 2019; Schwalbe et al., 2020). Datasets of personal health information are generated and controlled by a wide range of stakeholders, ranging from hospitals to doctors, digital health apps, and patients themselves (Blasimme et al., 2018). Data sharing is vital as innovators, such as pharma companies or digital health start-ups and big tech companies, require diverse health datasets to advance innovation, but lack multi-source health data that are typically available at health care institutions. Inter-stakeholder data sharing is thus needed to break down data silos and enable digital and personal health innovation (Landers et al., 2023).

Public private partnerships (PPPs) have been defined as “collaborative models based on a contractual agreement between at least one not-for-profit organization and at least one for-profit organization” (Stevens et al., 2013, p. 133), and are a particularly promising form of inter-stakeholder data sharing, as private and public entities each control distinct yet highly complementary resources (Ballantyne & Stewart, 2019, p. 6; Thorisson & Stein, 2003). Public and non-for-profit parties such as hospitals, public health agencies, government agencies, non-governmental organizations, and academia have access to unique research data and scientific and medical expertise. They typically follow a public mandate and are committed to advancing the public good as their highest priority. As a result, they enjoy high public trust, and are frequently entrusted as custodians of individual and public data (Ghafur et al., 2020). For-profit entities in the health domain, such as pharma, medical device, or biotech companies, understand market needs, and have drug and product development capabilities, and know-how in navigating regulatory pathways. These private companies control a considerable share of the development and delivery of healthcare products and services. It is typically private entities that transform research discoveries into products that improve patient welfare. PPPs are often formed to share complimentary resources and encourage partners to provide services that are societally beneficial (i.e., have positive externalities such as education).

In healthcare, PPP’s first major wave of dissemination occurred in the context of enhancing public health in low- and middle-income countries (LMICs) from the mid-1990s. They continue to arise for product development, disease control through product donation and distribution, or the general strengthening or coordination of health services (Widdus, 2001). Pharmaceutical companies alone, for example, possess core capabilities around product development, production, marketing and distribution, that were and are vital for tackling infectious diseases (e.g., malaria) in low- and middle-income countries (LMICs) (Widdus, 2005).

Over the last decade, PPPs expanded from their focus on infectious disease to become a major vehicle to advance drug development for the treatment of diseases and innovative approaches around personalized health (Davis et al., 2021). This was fueled by the realization that major innovations in healthcare, in particular personalized health or medical application of AI, will require the pooling of resources held by diverse stakeholders (de Vrueh & Crommelin, 2017). The growing importance of PPPs for drug development and modern health manifests itself in the considerable impact and growth of two major PPP umbrella organizations—the EU’s Innovative Health Initiative (IHI) and the US’ Critical Path Institute (C-Path). The IHI was formed between the European Federation of Pharmaceutical Industries and Associations (EEPIA), a major pharma body, and the EU in 2008. By 2021, the IHI had led to 144 projects with over 3,000 participants and combined budget of over five billion euros (Davis et al., 2021). Similarly, the United States’ Food and Drug Administration (FDA) initiated the Critical Path Initiative to “help modernize the development, evaluation, manufacture and use of FDA regulated products.” This has led to the creation of Critical Path Institute (C-Path), an independent, non-profit PPP in which the FDA and the European Medicine Agency (EMA) are partners. Thus far, 68 C-Path-related PPPs have led to core innovations in drug development and regulatory standards. The Critical Path for Alzheimers Disease (CPAD), for example, is a partnership between public bodies such as the FDA, the EMA, and the National Institute of Health (NIH), as well as most major pharmaceutical companies such as Roche, Novartis, GSK, Takeda, Biogen, or Merk—to name but a few. It has been claimed that this collaboration has become the “best data source in the world for exploring predictive modelling and understanding how patient selection and trial design impact Alzheimer’s drug development.” (Critical Path Institute, 2023) Indeed, over 350 institutions have shared more than 40,000 patient records from over 50 countries. The resulting impact is considerable: EMA and FDA, two of world’s most leading regulatory agencies, have adopted CPAD’s Clinical Trial Simulation for Mild-to-Moderate AD that helps to improve and assess clinical trial design. CPAD and C-Path thus help to considerably accelerate the increasingly lengthy and resource-intensive regulatory review of novel treatments and advance regulatory science. CPAD is just one example of where a health data PPP has had a considerable impact in ensuring that safe treatments reach patients faster.

Despite health data PPP’s considerable potential impact, they have also been the subject of controversy. Public and private partners’ often contradictory and competing incentives can endanger partnerships and may often lead to the appearance that the public’s and patients' interest may not be fully served. Given PPP’s unique value contributions, avoiding PPPs altogether would not serve the public interest. Therefore, addressing contradictory interests in PPPs poses a highly important business ethics problem.

In the setting of personalized health innovation, there is a need to resolve fundamental differences in views on health data. For public entities, data should be treated as a public good—it should be non-excludable (accessible to all), non-rivalrous, and should benefit the public (Malkin & Wildavsky, 1991). Private entities, however, often view data control as a source of competitive advantage—through making it exclusive, i.e., limiting access, they can derive a stronger competitive position and generate higher profitability. Private entities also have a fiduciary responsibility to their shareholders; they are legally required to prioritize shareholder interests and generate a profit. This difference in motivation marks the fundamental conflict between the goals of private and public organizations.

In addition to the financial aspect, considerable differences exist in terms of legitimacy. Public institutions often enjoy and benefit from what is known as a social license. Social license provides legitimacy for an organization (private or public) to use public resources and perform vital activities. This is derived from the public’s trust that these organizations’ use of resources, such as data, will ultimately serve the public interest and create public welfare (Xafis, 2015). In the context of PPPs, Ballantyne and Stewart (2019, p. 308) observe that “social license granted for data use in the public sector will not automatically extend to data sharing with the private sector.” Indeed, data donors’ willingness to share data with private organizations has declined since 2016 to a level significantly lower than for public institutions (Ghafur et al., 2020). In Switzerland, for example, pharmaceutical and health insurance companies have been found to be the institutions least trusted by the general public to use anonymized health data (Brall et al., 2022; Pletscher et al., 2022). Social license, or lack thereof, thus makes data sharing in public private partnerships challenging.

In order for PPPs to be successful, they must resolve these ethical issues and manage misaligned incentives in order to realize the potential of data sharing, and ultimately advance personalized medicine. To this end, ethical guidelines have been developed by government authorities, public organizations, and hospitals across different healthcare ecosystems. This paper thus reports on the PPP ethics guidelines developed in the Swiss Personalized Health Network (SPHN) in response to these important ethical challenges. We propose that considering these aspects of business and data ethics can considerably inform practice in a way that has relevance beyond Swiss health data PPPs. Health data sharing commonly fails to meet public expectations, both globally (Akhlaq et al., 2016; Tim Hulsen, 2020; Schwalbe et al., 2020) and in Switzerland (Vayena et al., 2018). Modern healthcare provision increasingly requires multi-stakeholder collaboration that has proven to be complex (Vayena, 2021). As such, the reported process and outcome constitute a case of translating ethics into practice through a culture of partnership around shared principles.

## Guideline Development Context

The authors’ observations in this paper are based upon insights gathered while the Swiss Personalized Health Network (SPHN) developed ethical guidelines for public private partnerships for health data. Established by the Swiss government to facilitate health data exchange, SPHN aims to establish coordinated data infrastructures to make health-related data available, interoperable, and shareable. Its ultimate goal is to accelerate innovation and research in personalized medicine, for the benefit of society (Vayena et al., 2018). Until 2020, SPHN-funded projects were primarily focused on cooperation among public sector institutions, in part due to a lack of guidance on how to collaborate with private organizations. Given the considerable potential benefits of collaboration with the private sector, and its ability to further the mission of SPHN, SPHN’s Ethical, Legal, and Social Implications advisory group (ELSI Advisory Group) was tasked with developing a PPP guidance document for negotiating and establishing PPPs involving data from SPHN-funded projects.

From November 2020 to November 2021, the ELSI Advisory Group developed the guidelines using a multi-stakeholder consultation methodology that allowed for an iterative process where stakeholders could reflect upon and share their respective incentives, values, and terminology. Before the initial guideline drafting, we conducted a literature search regarding ethical principles relevant to PPPs. Eight hospital representatives and the members of the ELSI Advisory Group also provided input on what they regard to be the core issues of health data PPPs. A preliminary version of the PPP guidelines was developed and the ELSI Advisory Group then solicited feedback from core SPHN stakeholders including health authorities, universities, university hospitals, and non-profit (research) organizations. Two stakeholder workshops, comprised of 34 stakeholders from five private companies and 16 public entities, served to validate and further develop the PPP guidance document. The SPHN National Steering Board was also involved in two rounds of consultations and endorsed the final version of the PPP guidance document during its meeting in September 2021. The final, approved, guidelines can be accessed at https://sphn.ch/document/guidance-on-ethical-health-data-sharing-in-public-private-partnerships/.

## Ethical Principles in PPPs

**Fig. 1:**
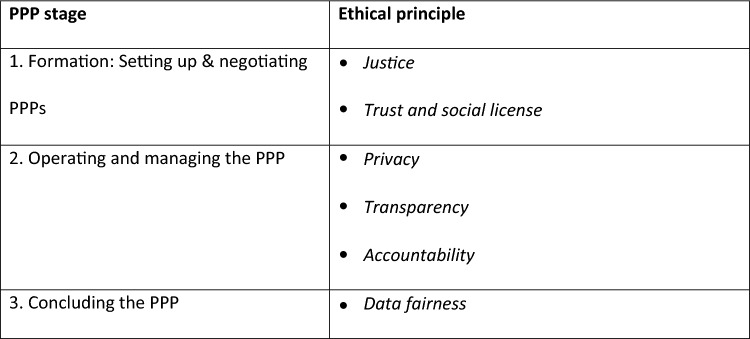
Core ethical principles for PPPs

Understanding ethical issues that arise in PPPs and identifying corresponding theoretical ethical principles was a foundational step in the SPHN guideline development process. These principles were justice, trust and social license, privacy, transparency, accountability, and data fairness. The principles are ordered according to their occurrence in a typical PPP lifecycle in Fig. 1 and described briefly in a way that highlights their relevance to PPPs hereafter.

- Justice is a fundamental principle of research ethics. It requires equal treatment, fairness, and inclusion for people participating in, or abstaining from, clinical research (Miracle, 2016). The advantages and burdens of research should be shared fairly (Gostin & Powers, 2006). Research should not involve groups who are unlikely to receive a benefit (US National Commission for the Protection of Human Subjects of Biomedical & Behavioral Research, 1978). Partners must determine ownership and exclusivity of data, and allocate value generated through a PPP. A frequent criticism of PPPs is that they “provide private partners the chance to appropriate public datasets or extract undue value from their access” (Ballantyne & Stewart, 2019, p. 319). From a public perspective, taxpayer funded and voluntarily donated public health data should remain a public good, and should benefit those who share it.
- Data fairness has gained attention as data have become an increasingly important innovation resource. Data fairness requires that data should be available to all. Organizations and individuals providing data should receive formal recognition, and data should be treated in accordance with the FAIR principles—making data findable, accessible, interoperable, and reusable (Mons, 2018; Wilkinson et al., 2016). Data fairness requires that data be made available at the end of a PPP for future research according to these principles.
- Privacy is a critical ethical issue in PPPs. A central question is whether data donors’ right to privacy is negatively affected when their data are shared with private sector entities. While health data are typically (pseudo)-anonymized and encrypted before it is shared, complete anonymity is hard to attain, and the techniques used to achieve it may reduce a dataset’s scientific value (Basso et al., 2016; Culnane et al., 2017; Scheibner et al., 2021). Privacy-enhancing technologies promise to resolve some of these issues, but considerable technological progress is still needed (Cha et al., 2019; Scheibner et al., 2021). A crucial dilemma thus emerges between protecting data donors’ privacy and optimizing publicly beneficial research outcomes.
- Transparency in the context of PPPs “might include making the data uses, expected benefits, … degree of security, … research results … accessible to the public” (Ballantyne & Stewart, 2019, p. 323). Transparency is of high moral importance when individuals are providing highly sensitive personal data without full knowledge of where their data might go. Depending on the circumstances, however, private and public partners may have an incentive to avoid full transparency. Private organizations have pointed out that transparency could make strategic information available to competitors and would thus be commercially disadvantageous. For public institutions, transparency can lead to greater public scrutiny, as PPPs can be contentious. Taking transparency a step further, accountability in health data PPPs requires sufficient transparency to enable data donors and the public to hold partners accountable for how they handle data. This implies that PPPs engage the public in the active governance of PPPs.
- Trust is a vital ethical issue in PPPs. Exchange of sensitive health data coincides with a parallel transfer of trust from the data provider (data subject, PPP institution) to the data receiver (private or public PPP institution). Trust implies a moral responsibility that the data receiver upholds such principles as data fairness and protection of confidentiality (Muller et al., 2021). In order for this transfer to take place, however, recipients need to be trusted. Organizations that have a high social license, for instance, are trusted by the public to use public resources to further a common good (Carter et al., 2015). Trust and social license are typically earned through a record of trustworthy behavior, although this may be somewhat distorted by a general public perception of an organization’s characteristics (e.g., industry association, for-profit status). When private or public partners lack social license, PPPs are usually complicated considerably (Grant et al., 2013).

## Applying Ethical Principles to the Development of PPP Guidelines

**Fig. 2:**
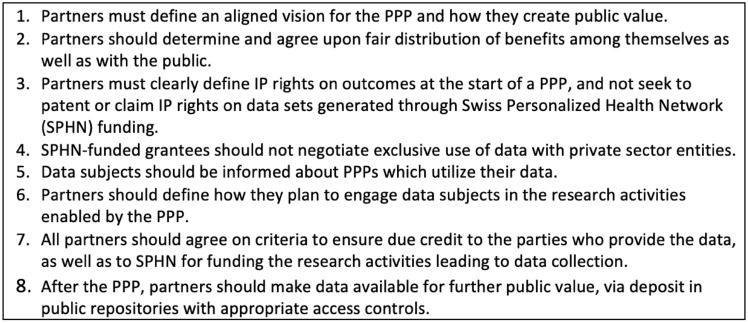
The SPHN PPP guidelines

The stakeholder consultation and review of ethical principles led to the identification of eight guidelines (Fig. 2) that constitute the core of the SPHN PPP guidance document. Taken together, the guidelines identify and propose resolutions to the primary ethical issues of health data sharing in PPPs. From a practical perspective, they can be conceived in four clusters: defining an aligned vision (Guideline 1), agreeing on a fair distribution of benefits (Guideline 2), determining data ownership (Guidelines 3, 4, and 8), and involving data subjects (Guidelines 5–7). In this section, we describe how individual ethical guidelines were developed through first analyzing how ethical issues arise in practice, and then identifying how ethical principles and theories can be applied to understand and practically resolve these issues.

- **Defining an aligned vision: Guideline 1**

**Table:** 

“1. Partners must define an aligned vision for the PPP and how it creates public value.”	

The first and arguably most important of the SPHN PPP guidelines requires partners to define a precise aligned vision at the beginning of the collaboration. This vision should identify a specific public good, related to public health, that both parties seek to meaningfully advance through a collaboration. The relevant ethical principles outlined above, in particular, trust, social license, and data fairness, play a crucial role here. Examples of a concrete aligned vision could be to advance a cure for a rare disease or develop novel genomics-based cancer testing. Research has shown that PPPs are more successful when a clear vision is identified and followed (Carol and Sang, 2008). Indeed, multi-stakeholder partnerships often struggle or fail as a result of the need to “navigate multi-level meaning systems” (Easter et al., 2022, p. 2). Each partner’s beliefs and attitudes shape their perspective on the purpose the partnership seeks to advance. Without intentionally sharing and aligning their incentives, partners may ultimately follow implicit individual incentives and pay mere lip service to superficially defined aims, assuming the partnership to be virtuous (Macdonald & Chrisp, 2005). Internally, defining an aligned vision therefore enables the parties to work towards the same direction and resolve potential misalignments. Externally, defining an aligned vision can strengthen the social license and accountability of the PPP. Recent surveys of the Swiss public as well as patient populations revealed “well-intentioned purpose” as the most important requirement for sharing their anonymized data for medical research (Pletscher et al., 2022). An aligned vision allows the broader public to better understand the societal contribution of a PPP, ensuring that public institutions do in fact further the common good, thereby supporting the principle of data fairness.

At PPP inception, defining an aligned vision around an opportunity in public health should be the first negotiation step, prior to addressing legal and commercial details. From there, “the aligned vision should help define the technical, legal, and commercial aspects of the PPP” (Swiss Personalized Health Network, 2021, p. 6). The impact of a PPP will largely depend on how well the aligned vision is defined and expressed in measurable steps, and how committed PPP partners are to its execution. To operationalize an aligned vision, key performance indicators (KPIs) should directly tie to the overall health priorities and clear use cases. Tracking these publicly can help ensure that the aligned vision is not mere lip service or ethics washing. Data uses beyond the aligned vision should require the mutually agreed revision of the PPP agreement.

Committing diverse stakeholders to an aligned vision and encouraging collective action has recently been advocated by business leaders, policy makers, and academics within and beyond healthcare. In her recent book *Mission Economy,* economist Mazzucato argues that pan-stakeholder collaboration focused on social missions (e.g., global warming, inequality) is vital for societal advances (Mazzucato, 2021). Business leaders have recently called for a pivot towards inclusive stakeholder capitalism whereby institutions not only optimize profits, but seek pan-stakeholder action to further the greater public good (Hunt et al., 2020; Schwab, 2021). The World Health Organization (WHO) has also called for what is essentially an aligned vision, rallying collective action from private and public stakeholders*.* The WHO Council on the Economics of Health For All seeks to improve global access to healthcare, clearly detailing measurement systems, capacity building, and innovation initiatives to promote effective collaboration among member organizations (World Health Organization, 2021). The real-world impact of an aligned vision will depend on the monitoring and enforcement of the partners’ commitments. Public transparency, particularly KPIs, can greatly enhance motivation to deliver on the aligned vision.

(b) **Fairly and pragmatically distributing benefits: Guideline 2**

**Table:** 

“2. Partners should determine and agree upon fair distribution of benefits among themselves as well as with the public.”	

This principle addresses the extensive debate around the measurement and allocation of the benefits of PPPs, linking back to the principles of fairness and justice, and invoking issues of trust and social license. Data are provided voluntarily by data subjects and collected by public bodies. The principle of data fairness implies a moral responsibility to allocate adequate value back to patients, the public, and those compiling the data. However, if PPPs are viewed as enriching private parties by sharing access to publicly funded resources, the social license of PPPs is weakened.

Measuring value creation (especially monetarily) and agreeing on value sharing is highly complex and requires considerable time and leadership resources. A range of models and valuation methodologies are proposed in the literature (Harwich & Lasko-Skinner, 2018). Examples include profit sharing, cost reimbursement for public institutions, and barring cooperation with profit-generating entities. Among the stakeholders that were consulted in our SPHN process, no consensus on a best practice emerged. Choice of model really varies on a case-by-case basis.

As such, the PPP guidance document recommends a pragmatic approach to distributing value within PPPs. Negotiating and tracking value sharing agreements is resource-intensive and inevitably imperfect (i.e., rarely perfectly equal). Excessive focus on how much monetary individual parties capture can even lead to termination of a partnership. Instead, partners should prioritize research progress that advances the common good. Health data PPPs often occur at early stages of the drug or product development process (pre-competitive or proof-of-concept PPPs), when data and knowledge generated are relatively fundamental. Before a financial value is assigned (e.g., the price of a drug), many more value-adding steps will occur (e.g., clinical trials), making it difficult to estimate the PPP’s share in the final value creation, something usually decided by a private firm. Similarly, defining and accounting for public value, much like accounting for private company value capture, may be imprecise.

Partners should thus strive to attain fair but pragmatic value distribution. This guideline holds that in the SPHN context, the main value of a PPP is to deliver responsible and innovative treatments that advance quality of life. It goes on to say that “data subjects should not expect compensation, and institutions should not expect a financial surplus beyond covering expenses” (Swiss Personalized Health Network, 2021, p. 7). Rather, the main compensation should be an advance in public and patient utility. Fair distribution is understood in a utilitarian rather than an egalitarian sense, while the latter implies that each party should receive the same bundle of material goods, utilitarian distribution prioritizes maximizing value to society (Lamont & Favor, 2017). This can be justified by both the ethical goal of maximizing societal over individual welfare, and the practical reality that calculating and distributing monetary value is highly burdensome, and with limited net welfare gain. Nevertheless, partners should be transparent about the value creation they hope to attain through a PPP. As no two PPPs are the same, the PPP guidance document does not advocate for one particular value sharing model; in certain circumstances distributing monetary value may be adequate and feasible.

(c) **Enabling fair access to data, IP, and research outcomes: Guidelines 3, 4, 8**

**Table:** 

“3. Partners must clearly define intellectual property (IP) rights on outcomes at the start of a PPP, and must not seek to patent or claim IP rights on data sets generated through SPHN funding. 4. SPHN-funded grantees should not negotiate exclusive use of data with private sector entities. 8. After the PPP, partners should make data available for further public value, via deposit in public repositories with appropriate access controls.”	

Taken together, guidelines 3, 4, and 8 discuss issues around data access and control that involve intellectual property and address the ethical principles of justice, data fairness, and fair allocation of benefits. Guideline 3 recommends that PPP parties determine intellectual property (IP) rights for joint products at the beginning of the PPP, and may not patent SPHN-funded datasets. Guideline 4 states that SPHN grantees may not grant exclusivity for SPHN-funded data to private partners. Guideline 8 details how data resulting from a PPP can be made available for further public use after the PPP.

IP and use rights in PPPs receive considerable attention, in PPPs and in the literature (Ballantyne & Stewart, 2019; Laverty & Poinot, 2014; Oguamanam, 2010). Differences between private and public values, incentive structures, and operating models are particularly apparent. Public organizations such as SPHN are committed to maximizing public access and value for datasets that they fund. Private companies often seek to restrict access, in order to be more competitive. The PPP guidance document resolves this conflict by promoting data fairness. Partners cannot place IP on SPHN-funded input datasets (Guideline 3). Exclusive use rights are also restricted (Guideline 4). The guidelines recommend, however, to allow IP on research outcomes that were mostly advanced by private actors. This appears reasonable in health data sharing contexts involving private parties, as IP is often necessary to incentivize private innovation.^1^ Similarly, data resulting from a PPP should be made available in publicly accessible repositories (Guideline 8), following the FAIR principles.

The PPP guidance designates SPHN-funded health data as a public good and seeks to uphold data fairness. Indeed, public value is maximized when data sources are freely combined. Recent research understands data sharing as inseparable from the research process (Blasimme et al., 2018; Gewin, 2016; Krumholz, 2015). Not sharing data has been described as “an impediment to the scientists of the future” (Milham, 2018, p. 2). Indeed, many countries’ IP laws forbid placing IP on research data. Making data available thus strengthens the social license of a PPP.

(d) **Involving and acknowledging data subjects: Guidelines 5–7**

**Table:** 

“5. Data subjects should be informed about PPPs which utilize their data. 6. Partners should define how they plan to engage data subjects in research activities enabled by the PPP. 7. All partners should agree on criteria to ensure due credit to the parties who provide the data, as well as to SPHN for funding the research activities leading to data collection.”	

Guidelines 5–7 address issues of transparency and accountability, emphasizing that data subjects should be adequately informed (5), engaged (6), and acknowledged (7) when data are shared. Before data collection, the informed consent process should inform data donors of how their data might be used in a PPP. At this time, they should also be informed where information about future uses of their data will be announced (e.g., by webpage link). The guidelines thus seek to enhance transparency while not constraining research. They address a core ethical dilemma in data donation: informing, engaging, and crediting individual donors are impossible when donor identities must remain anonymous. The guidelines nonetheless require partners to provide donors with transparency about who will use their data, for what purpose*,* and public good. In many circumstances individuals should and cannot be directly contacted for such purposes. Instead, information should be published on a dedicated PPP website, and by the institution that collected the data.

Guideline 6 addresses the role of data subjects as passive providers of data with limited means for accountability. The sheer number of data donors and their supposed lack of subject knowledge typically exclude them from more active roles in PPPs. Recent research, however, has shown the benefits of actively engaging patients, stakeholders, and data subjects in co-creation in health research and provision (Domecq et al., 2014; Osei-Frimpong et al., 2018; Vayena et al., 2016). Many research projects have addressed this by setting up patient and public involvement strategies (PPI). Indeed, a growing number of international and Swiss research organizations and funders require that PPI strategies be included in grants and research work (Colomer-Lahiguera et al., 2022; Swiss Clinical Trial, 2022).

PPP partners should define early on how they will engage with the public and organizations representing data donors. They should regularly consult these, actively engaging them in core decisions. As a result, accountability, social license, and pan-stakeholder learning will be enhanced. The PPP guidance document complements and extends beyond existing legislation, such as Switzerland’s Human Research Act (Swiss Confederation, 2011), urging PPPs to maximize transparency where possible. It also partially resolves the practical dilemma between preserving individual anonymity and providing maximum transparency. Combined, the three principles aim to increase public trust and grant PPP partners a public license. This is highly relevant as concerns around transparency rank particularly high in discourse around PPPs.

## Conclusion and Further Research

Here, we have reported on the development of ethical guidelines for private public partnership based on the foundational ethical principles of justice, trust, privacy, transparency, data accountability, and data fairness. The principles are intended as an ethical supplement to existing Swiss national regulations, and do not summarize or comment on legislation that research partners should consult independently. Since the release of the guidelines, they have already become an integral requirement of SPHN’s “Call for National Data Streams” and “Call for Demonstrator projects.”^2^ Additionally, a recent survey of the Swiss public on requirements for data-sharing confirmed the relevance of the principles put forward by the SPHN PPP guideline. Well-intentioned purpose (35%), anonymity (30%), and trust/transparent institutions (17%) were listed as the most important requirements (Pletscher et al., 2022). These mirror the SPHN guidelines, including the strong emphasis on defining an aligned vision, enhancing trust and transparency. It is, however, noteworthy that the SPHN guidelines do not stress anonymity as prominently, as privacy is widely covered by Swiss legislation^3^; this may be a significant difference if our approach were to be mirrored in other locations.

While it is too early to determine the guidelines’ impact and acceptance throughout the ecosystem at this stage, further research should continue to monitor and assess the direct and indirect impact of the guidelines. Changes in technology or acceptance of health data sharing may, for example, change the needs of such a guideline. For example, privacy-enhancing technologies could help address concerns such as privacy, but may subsequently raise issues around consent, explainability, or data (Scheibner et al., 2021). Public and professionals’ attitudes to health data sharing may also evolve, not least as initiatives such as the European Health Data Space may lead to wide-spread acceptance or rejection of health data sharing. It remains to be seen whether this might hinder acceptance of the guidelines.

We are hopeful that this report of practical ethics guideline development offers relevant learning for the application of business ethics theory in practice beyond the Swiss health data ecosystem. The need to translate ethics principles into business, innovation, and research practice is a major challenge and has become the subject of many papers and academic works, giving rise to the field of “translational ethics” (Bærøe, 2014; Cribb, 2010). In the context of digital health or AI in medicine, for instance, ethical principles have been articulated, but practitioners have not crossed the translational chasm of implementing these in practice (Landers et al., 2023; Trocin et al., 2021; Vayena et al., 2018). The guideline development reported herein serves as a case study of how ethical principles can inform context specific ethical guidelines. Rather than simply outlining abstract concepts, the guidelines are based on ethical theory that was then adapted to the practical context of the Swiss healthcare ecosystem to include extensive actionable recommendations on how to realize the guidelines at the various stages of a PPP. The iterative feedback and final endorsement by leading stakeholders constitute an initial validation of the principles’ translational relevance. While evaluating the guidelines’ final “translational success” will have to wait until the guidelines have been adopted and implemented by SPHN-associated PPPs for some time, the guidelines nevertheless constitute a considerable example of how ethical theory can inform practice (i.e., translational ethics) in health data sharing.

To our knowledge, neither of the two major PPP umbrella organizations for health data, C-Path and IHI, had publicly issued ethics guidelines by January 2023 (IHI helpdesk, personal communication, January 30, 2023; K. Swingle, personal communication, January 31, 2023). The principles and guideline development principles developed herein might also be of considerable relevance for the emerging European Health Data Space (EHDS). The EHDS seeks to provide individuals control over their electronic health data, while it also plans to make data available for health research, innovation, and policy making. Pharmaceutical companies can request data access if they pursue legitimate purposes, such as furthering scientific research, innovation, or developing algorithms (Ostojic & Pavlovic, 2022). Beyond intent of legitimate purposes, pharmaceutical companies and institutions collecting EHDS data should be held accountable and receive guidance on what legitimate purposes are and how they should practically be following. Developing and enforcing ethics guidelines seems highly advisable, not least since stakeholders’ trust in, and ultimate success of, the EHDS will depend on the public perception of such industry participations. We are thus hopeful that the approach, discussion of ethical principles and practical steps towards guideline development reported in this paper may be of help in this context. That said, it is again vital to stress that the principles outlined here must be adapted to the economic, social, and legal national implementation contexts.

As PPPs are widely utilized in sectors such as infrastructure or education, the insights of this paper might be further applicable to these contexts. Extensive commentary has emerged on the limits of, and even damage inflicted when PPPs are adopted in the wrong context (Gideon & Unterhalter, 2017; Leigland, 2018). The World Bank, a leading proponent of PPPs, has developed a range of resources to help partners validate whether a given PPP should be commenced at all (The World Bank Group, 2016). While these tools evaluate the macroeconomic, financial, and legal viability of PPPs, they offer less assistance in assessing ethical aspects. However, literature critically assessing PPPs extensively shows that even PPPs that are legally and financially viable on paper, may turn out to be destructive when parties are too focused on their own priorities (Torchia et al., 2015). Here, ethics guideline can help to reduce harm and prevent unjustified PPPs: defining an aligned vision, for instance, will likely reveal conflicts of interests. Aligning on PPP guidelines early may thus serve as a litmus test for potential partners: where values and priorities cannot be aligned, e.g., where profits are a sole motivator “crowding-out” any additional purposes, PPPs should not be formed. Future research should study such instances to further refine criteria for setting up PPPs and defining ethics guidelines across sectors.

Looking forward, PPPs—particularly in health data sharing—promise to deliver unique value and propel medicine forward. We propose that ethics guidelines can help to resolve PPPs’ ethical conflicts and address shortcomings from the beginning. We thus encourage PPPs across sectors to define ethics guidelines early by adopting the approach outlined in this paper to their specific context and needs.

## Data Availability

No additional relevant data or code was collected or developed for this study.

## Footnotes

1 According to SPHN legal agreement templates, IP may either be jointly owned by the parties or “jointly owned by the parties for common works and is otherwise owned by the party generating it.”

2 C.f. Call for National Data Streams https://sphn.ch/wp-content/uploads/2021/05/Call_2021_NDS.pdf and Call for Demonstrator projects: https://sphn.ch/wp-content/uploads/2022/05/Call_Demonstrator_NSB_final.pdf.

3 C.f. Federal Act on Data Protection (FADP) https://www.fedlex.admin.ch/eli/cc/1993/1945_1945_1945/en or Human Research Act https://www.fedlex.admin.ch/eli/cc/2013/617/en.
